# Trends in suicidal behavior at a general hospital emergency department in southern Brazil

**DOI:** 10.1590/2237-6089-2019-0080

**Published:** 2020-11-17

**Authors:** Betina Lejderman, Aline Parisotto, Lucas Spanemberg

**Affiliations:** 1 Núcleo de Formação em Neurociências Escola de Medicina Pontifícia Universidade Católica do Rio Grande do Sul Porto AlegreRS Brazil Núcleo de Formação em Neurociências, Escola de Medicina, Pontifícia Universidade Católica do Rio Grande do Sul (PUCRS), Porto Alegre, RS, Brazil.; 2 Unidade de Internação Psiquiátrica Hospital São Lucas PUCRS Porto AlegreRS Brazil Unidade de Internação Psiquiátrica, Hospital São Lucas, PUCRS, Porto Alegre, RS, Brazil.

**Keywords:** Suicide, suicidal ideation, suicide attempt

## Abstract

**Objective:**

To assess the prevalence of and factors associated with suicidal behavior in patients seen at the emergency department (ED) of a general hospital in southern Brazil.

**Method:**

Descriptive, observational, cross-sectional study. The records of all patients who had an emergency psychiatric consultation at the ED conducted by the emergency psychiatric consultation service at Hospital São Lucas da Pontifícia Universidade Católica do Rio Grande do Sul in 2016 and 2017 were analyzed and stratified by sex and by age groups (15-29 years, 30-49 years, 50-69 years, and 70 years and older). Suicidal behavior was characterized by factors such as thoughts of death, suicidal thoughts, and suicidal risk. Suicidal behavior was compared by sex and between age groups with chi-square tests. Multivariate analysis of suicidal behavior and gender, age, and specific diagnoses were compared with Poisson regression.

**Results:**

A total of 1,172 records from January 2016 to December 2017 were examined. There were more ED visits by females (63.1%) than males. Younger patients (15-29 years) had a higher severe risk of suicide than elderly (≥ 70 years) patients (54.1 vs. 19%; p < 0.01). Indicators of suicide behavior stratified by sex and by age group revealed marked differences between age groups for all variables among female patients. Overall, age group patterns for males were very similar in terms of suicidal behavior variables.

**Conclusions:**

A high prevalence of suicidal behavior was observed in this sample, particularly among young adults and especially associated with female gender and diagnoses of depression and personality disorders.

## Introduction

According to the World Health Organization (WHO), more than 800,000 people die by suicide each year.^1^ For every suicide, there are many more people who attempt suicide every year and there are indications that for each adult who died of suicide there may have been more than 20 others attempting suicide; significantly, a prior suicide attempt is the single most important risk factor for suicide in the general population.^1^ Suicidal behavior ranges from ideation of killing oneself to forming a plan and obtaining the means to execute the act. Among young people 15-29 years of age, suicide is the second leading cause of death globally,^1^ and the fourth leading cause of death in Brazil.^2^ The global suicide rate is 10.6 per 100,000 people.^3^ The mortality rate from suicide is rising in Brazil, from 5.3 per 100,000 in 2011 to 5.7 per 100,000 in 2015.^2^ These rates are higher in the southern states of Brazil. In 2016, Rio Grande do Sul, the state with the highest incidence of suicide, had a rate that was double the Brazilian average.^4^ Regardless of the large variability in average suicide rates among Brazilian states, there was a stable or increasing trend in suicide rates from 1997 to 2015 in Brazil.^5^

Suicidal ideation and fatal and nonfatal suicide attempts often begin at age 12-13 and increase during adolescence,^6^ and may establish a pattern of suicidal behavior in young adults.^7^ Suicidal behavior among adolescents and young adults is related to this stage of development, which is characterized by presence of risk factors for suicide such as substance abuse, depression, thrill-seeking, and high-risk behaviors.^8^

The hospital ED is often the gateway to care for unrecognized or untreated cases of mental disorders, including suicide attempts and patients at risk of suicide.^9 , 10^ The risk of suicide or other causes of death following ideation has not been well established. A recent meta-analysis found that suicidal ideation was associated with more than a three-fold increase in the risk of suicide,^11^ but the authors also noted considerable between-study heterogeneity. A significant number of individuals who complete suicide have had contact with the health system in the month prior to death.^12^ The first contact with the health system can offer an excellent opportunity to identify patients at risk and to intervene in potentially fatal cases. In a multicenter study of emergency department patients with elevated suicide risk, a combination of brief interventions administered both during and after the emergency department visit decreased suicidal behavior after discharge.^13^

Despite the importance of and recent increase in suicidal behavior, there is a dearth of studies investigating the prevalence of suicidal behavior in emergency care settings in Brazil. This study aims to describe the prevalence of suicidal thoughts and behaviors in people presenting to the ED at a general hospital in southern Brazil and investigate associations with clinical and sociodemographic variables.

## Materials and methods

### Design and sample

This is a descriptive, observational, cross-sectional study. The records of all patients who had an emergency psychiatric consultation at the Hospital São Lucas emergency department (HSL ED), affiliated with the Pontifícia Universidade Católica do Rio Grande do Sul over a two-year period (January 2016 to December 2017) were analyzed and stratified by sex and by age groups (15-29 years, 30-49 years, 50-69 years, and 70 years and older). Patients under the age of 15 were excluded because they constitute a specific population and there is no specialist pediatric mental health service at the HSL. In total, 1,172 records were analyzed.

### Setting

All patients presenting at the HSL ED are initially screened by a general practitioner. The psychiatric emergency consultation service is called when an urgent need for psychiatric care is identified, such as suicidal behavior, psychomotor agitation, or risk of aggression. The service is staffed by on-call physicians from the HSL residency and fellowship programs in psychiatry. All consultations are entered into the medical record system and the management plan is discussed with the attending physician.

### Instrument

The psychiatric emergency assessment record is routinely used by the HSL psychiatric emergency consultation service as an interview script and on-call log. It is completed by the on-call physician in charge of urgent psychiatric consultation at the time. The record covers 10 items: patient identification and sociodemographic data; reason for evaluation; suicide risk assessment; presence of clinical comorbidities; current or past smoking; use of psychotropic drugs; chemical dependency; history of follow-up by mental health professionals; current medical management; and diagnostic hypothesis. Suicide risk is assessed with the Mini International Neuropsychiatric Interview (MINI), version 5.0.^14^ The MINI is a brief standardized diagnostic interview used in clinical practice and research in primary care and psychiatry. To assess the risk of suicide, the patient is asked whether, during the last month, they felt they would be better off dead or wanted to be dead; if they meant to harm themselves; if they had ever thought about suicide; if they had thought of a way to attempt suicide; and if they had made a suicide attempt; and also if, at any point in their lives, they had made a suicide attempt. Each affirmative answer is scored. The scores are added to define the patient’s risk of suicide as none (0), low (1-5), moderate (6-9), or high (≥ 10). Finally, the patient is asked about family history of completed suicide.

### Statistical analysis

All analyses were performed using the Statistical Package for the Social Sciences (SPSS). For descriptive analysis, continuous variables were presented as mean (standard deviation [SD]), and categorical variables as n (%). Between-group differences in continuous variables were analyzed with a t-test for independent samples. Categorical variables were analyzed using the chi-square test. Residuals were adjusted to reveal the differences between categories in each variable. The significance level was set at p < 0.05. For multivariate analyses of suicidal behavior and gender, age, and specific diagnoses, regressions and prevalence ratios were calculated with robust variance. Poisson regression with robust variance was used for these analyses.

### Ethical aspects

This study was approved by the institutional review board at Pontifícia Universidade Católica do Rio Grande do Sul. Data confidentiality was ensured under the terms of a data use agreement. The study protocol is registered in the Brazilian research ethics database under no. CAAE 90002218.0.0000.5336.

## Results

A total of 1,172 records from consultations between January 2016 and December 2017 were examined. Mean (SD) age was 38.6 (±18.1) years. There were more ED visits by females than males: 63.1% of the sample. The most frequent primary complaints in the sample were suicidal ideation (20.0%) and suicide attempt (17.7%). The most common psychiatric diagnosis was unipolar depression (34.7%), followed by bipolar disorder (16.7%). Table 1 presents the sociodemographic characteristics for the entire sample and stratified by age ranges.

**Table 1:** Clinical and sociodemographic characteristics for the whole sample and each age group: HSL ED, 2016 and 2017

Variables	Total sample	15-29 years	30-49 years	50-69 years	≥ 70 years
Gender female (n = 1,172)	739 (63.1)	261 (61.0)	285 (68.0)	138 (56.3)	55 (68.8)
Age (n = 1,172)	38.6 ± 18.1	20.5 ± 4.0	38.5 ± 5.5	57.6 ± 5.7	78.2 ± 5.9
Marital status (n = 1,121)					
Single	494 (44.1)	344 (83.5)	111 (27.5)	32 (13.8)	7 (9.6)
Married/cohabitating	448 (40.0)	61 (14.8)	226 (55.9)	132 (56.9)	29 (39.7)
Separated/divorced	110 (9.8)	4 (1.0)	54 (13.4)	42 (18.1)	10 (13.7)
Widowed	69 (6.2)	3 (0.7)	13 (3.2)	26 (11.2)	27 (37.0)
Years of education (n = 983)	11.2 ± 3.8	10.9 ± 2.7	11.9 ±3.7	11.0 ± 4.8	9.9 ± 5.1
Occupation (n = 1,082)					
Active/employed	691 (63.9)	321 (80.5)	259 (66.4)	94 (42.0)	17 (24.6)
Unemployed	175 (16.2)	68 (17.0)	68 (17.4)	35 (15.6)	4 (5.8)
Retired	148 (13.7)	3 (0.8)	22 (5.6)	76 (33.9)	47 (68.1)
Transient disability	68 (6.3)	7 (1.8)	41 (10.5)	19 (8.5)	1 (1.4)
Chief complaint (n = 1,152)					
Suicidal ideation	230 (20.0)	90 (21.5)	87 (21.0)	48 (19.9)	5 (6.4)
Suicide attempt	204 (17.7)	107 (25.6)	70 (16.9)	23 (9.5)	4 (5.1)
Depressive symptoms	162 (14.1)	40 (9.6)	67 (16.1)	35 (14.5)	20 (25.6)
Anxious symptoms	138 (12.0)	44 (10.5)	65 (15.7)	25 (10.4)	4(5.1)
Psychotic symptoms	133 (11.5)	53 (12.7)	40 (9.6)	28 (11.6)	12 (15.4)
Altered behavior*	136 (11.8)	41 (9.8)	27 (6.5)	42 (17.4)	26 (33.3)
Drug use/misuse	77 (6.7)	23 (5.5)	33 (8.0)	19 (7.9)	2 (2.6)
Manic symptoms	29 (2.5)	6 (1.4)	9 (2.2)	11 (4.6)	3 (3.8)
Other	43 (3.7)	14 (3.3)	17 (4.1)	10 (4.1)	2 (2.6)
Primary diagnosis (n = 1,103)					
Substance use/misuse	112 (10.2)	41 (10.1)	41 (10.5)	27 (11.5)	3 (4.0)
Psychotic disorder	105 (9.5)	40 (9.9)	32 (8.2)	24 (10.3)	9 (12.0)
Bipolar disorder	184 (16.7)	38 (9.4)	70 (17.9)	60 (25.6)	16 (21.3)
Unipolar depression	383 (34.7)	125 (30.9)	145 (37.2)	87 (37.2)	26 (34.7)
Neurotic/anxiety disorder	157 (14.2)	66 (16.3)	61 (15.6)	24 (10.3)	6 (8.0)
Personality disorder	94 (8.5)	62 (15.3)	29 (7.4)	3 (1.3)	-
Other	68 (6.2)	32 (7.9)	12 (3.1)	9 (3.8)	15 (20.0)

Data presented as n (%) or mean ± standard deviation.

HSL ED = Hospital São Lucas emergency department.

* Includes aggressiveness and psychomotor agitation.

When the total sample was stratified by age group, statistically significant differences were found in almost all items indicative of suicidal behavior, with the exception of family history of suicide. In general, younger patients (15-29 years) had a higher risk of suicidal behavior than elderly patients. Considering the age groups analyzed, 61.0% of people aged 15-29 had suicidal thoughts, followed by 51.5% of people aged 30-49, 43.5% of people aged 50-69, and 20.3% of people aged 70 years and over. In the 15-29 age group, 35.2% had attempted suicide in the previous month, followed by 29.4% of people aged 30-49, 17.2% of people aged 50-69, and 13.9% of people aged 70 years and over. Differences in suicidal behavior by age group are described in Table 2 .

**Table 2:** Differences in suicidal behavior by age group: HSL ED, 2016 and 2017

	Total sample	15-29 years	30-49 years	50-69 years	≥ 70 years	p
Thoughts of death	634 (55.7)	261 (62.6)^3.6^	235 (58.3)	111 (46.4)^-3.2^	27 (34.2)^-4.0^	**< 0.01**
Want to hurt yourself	530 (46.5)	241 (57.7)^5.7^	185 (45.9)	87 (36.4)^-3.5^	17 (21.5)^-4.6^	**< 0.01**
Suicidal thoughts	582 (51.1)	255 (61.0)^5.1^	207 (51.5)	104 (43.5)^-2.7^	16 (20.3)^-5.7^	**< 0.01**
Thinking about a way to kill yourself	474 (41.8)	208 (49.9)^4.2^	175 (43.8)	79 (33.1)^-3.1^	12 (15.2)^-5.0^	**< 0.01**
Suicide attempt	317 (27.9)	147 (35.2)^4.2^	118 (29.4)	41 (17.2)^-4.2^	11 (13.9)^-2.9^	**< 0.01**
Lifetime suicide attempt	389 (34.6)	160 (38.6)^2.2^	148 (37.4)	65 (27.4)^-2.6^	16 (20.5)^-2.7^	**< 0.01**
Risk of suicide						
None	414 (36.4)	119 (28.5)^-4.2^	135 (33.7)	113 (47.3)^3.9^	47 (59.5)^4.4^	**< 0.01**
Slight risk	138 (12.1)	41 (9.8)	57 (14.2)	25 (10.5)	15(19.0)	
Moderate risk	76 (6.7)	32 (7.7)	27 (6.7)	15 (6.3)	2 (2.5)	
Severe risk	509 (44.8)	226 (54.1)^4.8^	182 (45.4)	86 (36.0)^-3.1^	15 (19.0)^-4.8^	
Family history of suicide	96 (9.4)	42 (11.4)	31 (8.4)	19 (8.7)	4 (5.9)	0.34

Data presented as n (%).

HSL ED = Hospital São Lucas, emergency department.

Superscript values denote residuals adjusted for post-hoc differences.

Bold type denotes p < 0.05, analyzed by the chi-square test.

Analysis of differences in indicators of suicide behavior stratified by sex and by age group revealed marked differences between age groups for all variables among female patients. For severe suicide risk, the 15-29 years age group were at higher risk of suicide than the 50-69 years and 70 years and over age groups (60.5 vs. 38.2% vs. 13.0%, p < 0.001). The lifetime suicide attempt rate was 44.7% among females aged 15-29 years, 41.8% among females aged 30-49, 30.4% among females aged 50-69, and 18.5% among females aged 70 years and over (p < 0.001 for 15-29 years vs. 50-69 and 70 years and over). The age group patterns for males were overall very similar in variables of suicidal behavior. The lifetime suicide attempt rate was 28.9% among males aged 15-29 years, 28.1% among males aged 30-49, 23.5% among males aged 50-69, and 25.0% among males aged 70 years and over. The results stratified by sex are shown in Table 3 .

**Table 3:** Differences in suicide behavior between age groups, stratified by sex: HSL ED, 2016 and 2017

	Females						Males					

	total n	15-29 years	30-49 years	50-69 years	≥ 70 years	p	total n	15-29 years	30-49 years	50-69 years	≥ 70 years	p
Thoughts of death	438 (60.7)	183 (70.9)^4.2^	170 (62.0)	67 (49.3)^-3.0^	18 (33.3)^-4.3^	**< 0.01**	196 (47.1)	78 (49.1)	65 (50.4)	44 (42.7)	9 (36.0)	0.42
Want to hurt yourself	366 (50.7)	168 (65.1)^5.8^	135 (49.3)	53 (39.0)^-3.0^	10 (18.5)^-4.9^	**< 0.01**	164 (39.3)	73 (45.6)	50 (38.8)	34 (33.0)	7 (28.0)	0.13
Suicidal thoughts	400 (55.4)	179 (69.4)^5.6^	150 (54.7)	63 (46.3)^-2.4^	8 (14.8)^-6.2^	**< 0.01**	182 (43.8)	76 (47.5)	57 (44.5)	41 (39.8)	8 (32.0)	0.39
Thinking about a way to kill yourself	323 (44.8)	141 (54.9)^4.0^	130 (47.4)	46 (33.8)^-2.9^	6 (11.1)^-5.2.^	**< 0.01**	151 (36.5)	67 (41.9)	45 (35.7)	33 (32.0)	6 (24.0)	0.20
Suicide attempt	214 (29.8)	97 (37.7)^3.5^	88 (32.4)	24 (17.6)^-3.4^	5 (9.3)^-3.4.^	**< 0.01**	103 (24.6)	50 (31.1)	30 (23.3)	17 (16.5)	6 (24.0)	0.06
Lifetime suicide attempt	277 (38.9)	114 (44.7)^2.4^	112 (41.8)	41 (30.4)^-2.3^	10 (18.5)^-3.2.^	**< 0.01**	112 (27.1)	46 (28.9)	36 (28.1)	24 (23.5)	6 (25.0)	0.79
Risk of suicide												
None	225 (31.2)	51 (19.8)^-4.9^	83 (30.3)	60 (44.1)^3.6^	31 (57.4)^4.3.^	**< 0.01**	189 (45.5)	68 (42.5)	52 (40.9)	53 (51.5)	16 (64.0)	0.19
Slight risk	97 (13.4)	28 (10.9)	40 (14.6)	15 (11.0)	14 (25.9)^2.8^		41 (9.9)	13 (8.1)	17 (13.4)	10 (9.7)	1 (4.0)	
Moderate risk	49 (6.8)	23 (8.9)	15 (5.5)	9 (6.6)	2 (3.7)		27 (6.5)	9 (5.6)	12 (9.4)	6 (5.8)	-	
Severe risk	351 (48.6)	156 (60.5)^4.8^	136 (49.6)	52 (38.2)^-2.7^	7 (13.0)^-5.4^		158 (38.1)	70 (43.8)	46 (36.2)	34 (33.0)	8 (32.0)	
Family history of suicide	61 (9.5)	29 (13.0)^2.2^	20 (8.0)	8 (6.4)	4 (8.9)	0.15	35 (9.2)	13 (9.0)	11 (9.2)	11 (11.8)	-	0.38

Data presented as n (%).

HSL ED = Hospital São Lucas emergency department.

Bold type denotes p < 0.05, analyzed by the chi-square test.

Univariate analyses were also conducted of associations between variables related to suicide risk and International Classification of Diseases, 10th revision (ICD-10). Major depressive disorder and personality disorders were associated with all variables related to suicidal behavior, with the exception of family history of suicide.

Multivariate analysis adjusted for gender, unipolar depression, and age indicated that female gender, young age, and a diagnosis of depression are risk factors for suicidal behavior. A significant association with female gender was found in half of the variables associated with suicidal behavior: thoughts of death, wanting to hurt oneself, lifetime suicide attempts, and severe suicide risk. There was no association between family history of suicide and suicidal behavior. Multivariate analysis adjusted for gender, personality disorder, and age revealed that female gender, young age, and diagnosis of personality disorder were risk factors for almost all variables related to suicidal behavior, except for thinking about a way to kill oneself and current suicide attempt, which were not associated with gender. Once more, there was no association between family history of suicide and suicidal behavior ( Table 4 ).

**Table 4:** Multivariate analysis of associations between variables associated with suicidal behavior and gender, age, and specific diagnoses (unipolar depression and personality disorder): HSL ED, 2016 and 2017

	Unipolar depression			Personality disorder		
	
	PR	95%CI	p	PR	95%CI	p
Thoughts of death						
Female	1.16	1.04-1.30	**< 0.01**	1.24	1.10-1.39	**< 0.01**
Unipolar depression	1.73	1.57-1.91	**< 0.01**	-	-	-
Personality disorder	-	-	-	1.32	1.17-1.49	**< 0.01**
Age	0.99	0.98-0.99	**< 0.01**	0.99	0.99-0.99	**< 0.01**
Want to hurt yourself						
Female	1.16	1.01-1.33	**0.03**	1.23	1.07-1.41	**< 0.01**
Unipolar depression	1.80	1.60-2.03	**< 0.01**	-	-	-
Personality disorder	-	-	-	1.41	1.22-1.62	**< 0.01**
Age	0.99	0.98-0.99	**< 0.01**	0.99	0.98-0.99	**< 0.01**
Suicidal thoughts						
Female	1.12	1.00-1.27	0.06	1.21	1.07-1.37	**< 0.01**
Unipolar depression	1.90	1.71-2.11	**< 0.01**	-	-	-
Personality disorder	-	-	-	1.32	1.16-1.51	**< 0.01**
Age	0.99	0.98-099	**< 0.01**	0.99	0.98-0.99	**< 0.01**
Thinking about a way to kill yourself						
Female	1.08	0.93-1.25	0.33	1.16	1.00-1.35	0.05
Unipolar depression	1.98	1.73-2.26	**< 0.01**	-	-	-
Personality disorder	-	-	-	1.42	1.20-1.68	**< 0.01**
Age	0.99	0.98-0.99	**< 0.01**	0.99	0.98-0.99	**< 0.01**
Suicide attempt						
Female	1.06	0.87-1.30	0.55	1.16	0.95-1.42	0.15
Unipolar depression	2.10	1.75-2.52	**< 0.01**	-	-	-
Personality disorder	-	-	-	1.35	1.06-1.74	**0.02**
Age	0.98	0.98-0.99	**< 0.01**	0.98	0.98-0.99	**< 0.01**
Lifetime suicide attempt						
Female	1.30	1.08-1.55	**< 0.01**	1.37	1.14-1.65	**< 0.01**
Unipolar depression	1.76	1.50-2.06	**< 0.01**	-	-	-
Personality disorder	-	-	-	1.48	1.20-1.82	**< 0.01**
Age	0.99	0.98-0.99	**< 0.01**	0.99	0.98-0.99	**< 0.01**
Severe suicidal risk						
Female	1.13	0.98-1.30	**0.10**	1.22	1.05-1.40	**< 0.01**
Unipolar depression	1.92	1.70-2.17	**< 0.01**	-	-	-
Personality disorder	-	-	-	1.34	1.14-1.58	**< 0.01**
Age	0.99	0.98-0.99	**< 0.01**	0.99	0.98-0.99	**< 0.01**
Family history of suicide						
Female	0.92	0.61-1.38	0.67	0.91	0.60-1.37	0.65
Unipolar depression	1.10	0.73-1.65	0.65	-	-	-
Personality disorder	-	-	-	1.43	0.76-2.69	0.27
Age	0.99	0.98-1.00	0.42	0.99	0.98-1.00	0.56

95%CI = 95% confidence interval; HSL ED = Hospital São Lucas emergency department; PR = prevalence ratio.

Bold type denotes p < 0.05; analyzed by Poisson regression.

## Discussion

The objective of this study was to analyze clinical and sociodemographic differences associated with suicidal behavior in patients seen at the ED by an emergency psychiatric consultation service at a general hospital. The results showed a high prevalence of suicidal behavior, most strongly associated with younger age, female gender, and diagnoses of depression and personality disorders. Approximately half of the subset aged 15-29 had suicidal thoughts and one-third of these young people had attempted suicide at least once before. Studies conducted in emergency departments in Brazil confirm the predominance of suicide attempts in the young population, especially among women.^15 - 17^ Since one of the main demands of psychiatric emergency consultation services is suicidal behavior, the fact that there are high rates of suicidal ideation and suicide attempt in the sample is unsurprising. We nevertheless highlight the finding that one third of the youngest group of patients had attempted suicide at least once before. In a previous study investigating emergency psychiatric care at our facility, the number one specific reason for psychiatric care among children and adolescents was suicide attempts.^18^

In our study, suicidal behavior was mainly associated with young age. The highest prevalence of severe suicide risk was identified in the 15-29 years age group. This age group also had higher prevalence rates of suicide attempts both in the last month and lifelong. A study conducted in emergency departments in the United States also found higher incidence of suicide attempts among women and individuals aged 15-19.^19^

Between 2011 and 2016, 69.0% of reported suicide attempts in Brazil were by women and the suicide attempts by women predominate in adolescence (10-19 years) and young adulthood (20-39 years).^20^ However, the risk of suicide is approximately four times greater in males. There is a discrepancy between the number of suicides and attempted suicides, with one possible explanation being the preferred methods of each gender: despite making more suicide attempts, women use less effective methods than men.^21^ This difference in behavior between the genders may explain the higher prevalence of women presenting to emergency departments,^15 , 16 , 22^ coinciding with our sample – in which female patients presented with a higher prevalence of suicidal behavior.

Studies have shown an association between mental illness and suicide.^23 , 24^ In our sample, the main mental health diagnosis was unipolar depression, which is one of the leading risk factors for suicide. Depressed patients are 20 times more likely to commit suicide than the general population.^25^ Depression is increasing among adolescents and young adults, especially among females.^26^ A recent study demonstrated increases in suicide rates since 2006.^27^ Self-inflicted injuries in adolescence may be indicative of the development of borderline personality disorder.^28^ A survey of young people in the United States (aged 10 to 24) between 2001 and 2015 showed an increase in the rate of females presenting to emergency departments with self-inflicted injuries from 2008 to 2015.^29^ In our sample, a diagnosis of a personality disorder was a risk factor for suicidal behavior. Thus, it is important to detect mental disorders and investigate suicidal behavior in all psychiatric disorders, especially when depression and personality disorder are in the differential diagnosis.

Many patients who seek care for nonpsychiatric complaints at emergency departments have undiagnosed mental illness.^30^ One study has shown that although suicidal ideation is frequent in adolescents seen in emergency departments, it goes unnoticed by physicians and patients are not referred to mental health services.^31^ Screening youth for suicidal behavior is an important way of preventing suicide.^32^ Use of standard screening tools in primary care increases detection of suicidal ideation, enabling referral to mental health services before a serious or fatal suicide attempt occurs.^33^ Effective intervention for suicide prevention includes involvement of family members and caregivers to provide young people with the necessary support and ensure commitment to follow-up after ED discharge.^34^ It is equally important to continue investigating suicidal behavior during such follow-up.^35^

This study has some limitations. The psychiatric emergency assessment record is completed by an on-call physician, and its items focus on delivery of urgent care; other information that is potentially useful from a research standpoint is not collected, and some records are incomplete. Since data collection occurred in a naturalistic environment, information such as the diagnostic hypothesis was obtained through a clinical interview (i.e., without the aid of psychometric instruments) and at a single point in time, and is thus susceptible to recall and information bias. Furthermore, data collection took place in a non-psychiatric department of a general teaching hospital that mostly serves patients with private health insurance and the findings are not therefore generalizable.

The hospital does not have a specialized pediatric psychiatry service and all potential psychiatric patients (including children and adolescents) are seen by professionals trained in general psychiatry. Furthermore, as a matter of course, the population sample was limited to patients who sought care at HSL.

## Conclusions

We found a high prevalence of suicidal behavior in this sample of patients seeking emergency psychiatric consultation at a general hospital, particularly among young women. Suicidality was associated mainly with young age and with diagnoses of depression and personality disorders. These findings highlight the need for robust assessment in general emergency departments, training of emergency department staff (including in the use of standardized tools to screen for suicidal behavior), and referral to specialized mental health services when needed.
